# Neomorphosis and heterochrony of skull shape in dog domestication

**DOI:** 10.1038/s41598-017-12582-2

**Published:** 2017-10-18

**Authors:** Madeleine Geiger, Allowen Evin, Marcelo R. Sánchez-Villagra, Dominic Gascho, Cornelia Mainini, Christoph P. E. Zollikofer

**Affiliations:** 1 Paläontologisches Institut und Museum, Universität Zürich, Karl-Schmid-Strasse 4, 8006 Zurich, Switzerland; 2 Department of Zoology, University of Cambridge, Downing Street, Cambridge, CB2 3EJ United Kingdom; 3 Institut des Sciences de l’Évolution - Montpellier, UMR 5554 CNRS, Université de Montpellier, EPHE, IRD 226, Cirad 2, Place Eugène Bataillon, 34095 Montpellier, cedex 05 France; 4 Department of Archaeology, Classics and Egyptology, University of Liverpool, 12-14 Abercromby Square, Liverpool, L69 7WZ United Kingdom; 5 Institut für Rechtsmedizin, Universität Zürich, Winterthurerstrasse 190/52, 8057 Zurich, Switzerland; 6 Tierpark Bern, Dählhölzli & Bärenpark, Tierparkweg 1, 3005 Bern, Switzerland; 7 Anthropologisches Institut und Museum, Universität Zürich, Winterthurerstrasse 190, 8057 Zurich, Switzerland

## Abstract

The overall similarity of the skull shape of some dog breeds with that of juvenile wolves begs the question if and how ontogenetic changes such as paedomorphosis (evolutionary juvenilisation) played a role in domestication. Here we test for changes in patterns of development and growth during dog domestication. We present the first geometric morphometric study using ontogenetic series of dog and wolf crania, and samples of dogs with relatively ancestral morphology and from different time periods. We show that patterns of juvenile-to-adult morphological change are largely similar in wolves and domestic dogs, but differ in two ways. First, dog skulls show unique (neomorphic) features already shortly after birth, and these features persist throughout postnatal ontogeny. Second, at any given age, juvenile dogs exhibit skull shapes that resemble those of consistently younger wolves, even in dog breeds that do not exhibit a ‘juvenilized’ morphology as adults. These patterns exemplify the complex nature of evolutionary changes during dog domestication: the cranial morphology of adult dogs cannot simply be explained as either neomorphic or paedomorphic. The key to our understanding of dog domestication may lie in a closer comparative examination of developmental phases.

## Introduction

Investigating how novel variation of domesticated forms is generated through changes in development and growth brings new insights into the micro-evolutionary processes of domestication. How do new morphological and behavioural peculiarities of domesticated species arise^1–4^? The retention of juvenile characteristics of wolves (*Canis lupus*) into the adulthood of domestic dogs has been suggested to describe the anatomical and behavioural disparity between the ancestor and the domesticated form^5–11^. Morphologically, the relatively short face and broad skull of domestic dogs have been interpreted as a paedomorphic pattern^5–10^. The size relationships between major portions of the skull during growth, coupled with a body size decrease due to domestication, could contribute to a juvenilized skull shape in domestic dogs^12^: the neurocranium scales negatively allometrically and the facial region positively allometrically with body size, thereby leading to a relatively short snout and proportionally large braincase in some domestic dogs. However, the paedomorphosis hypothesis has been challenged on various grounds^12–15^. In particular, a recent study revealed no resemblance between adult cranial shape of modern breeds and neither adult or juvenile wolves^15^. This led to the conclusion that dog cranial shape is neomorphic, i.e., showing novel, taxon-specific features that cannot simply be explained as paedomorphic (i.e., juvenilized) variants of the wolf morphology.

Neomorphosis and paedomorphosis as patterns of evolutionary-developmental differentiation between groups are not mutually exclusive but likely act in concert to generate taxon-specific morphology. Neomorphosis largely corresponds to the notion of heterotopy (originally coined by Haeckel^16^), which denotes modification of spatial/structural aspects of the ancestral developmental program leading to new morphology. Paedomorphosis, on the other hand, results from heterochrony, which denotes modification of temporal characteristics (onset, rate, and cessation) of the ancestral developmental program. Both empirical and theoretical data indicate that the evolutionary diversification of closely related taxa typically results from a combination of spatial and temporal modifications of the ancestor’s developmental program^17^. In hominoids, for example, neonates already exhibit distinct, taxon-specific cranial shapes, indicating prenatal neomorphosis^18^. Postnatal cranial ontogeny, on the other hand, largely unfolds along a trajectory shared by all taxa, while taxon-specific differences arise through heterochronic shifts along the trajectory^18^.

Here, we assess the relative contribution of neomorphic and paedomorphic modifications during dog domestication. Obviously, this can only be done by comparing patterns of ontogeny in wolves (the ancestral form) and dogs (‘descendant’ forms resulting from domestication). In this study, we test the neomorphosis and paedomorphosis hypotheses taking into account two aspects which have not yet been considered in similar studies: (1) comparing cranial ontogeny of wolves and dogs including juveniles and adults of both groups; (2) taking into account that different selection regimes may have acted on dog crania during the course of domestication. To track group-specific ontogenetic trajectories, we document cranial growth and development from early postnatal life to adulthood in cross-sectional ontogenetic series, using methods of geometric mophometrics^19,20^ and phenotypic trajectory analysis^21^.

Phenotypic trajectory analysis is a method based on geometric morphometrics^22^, which provides a straightforward operationalization of the concepts of heterotopic (neomorphic) and heterochronic (e.g., paedomorphic) modification of ontogenetic trajectories^23,24^. In our case, each group’s (wolf, dog groups and breeds) pattern of three-dimensional cranial shape change from juveniles to adults is characterized by an ontogenetic trajectory through multidimensional shape space. Provided that group-specific trajectories are fairly linear, their directions can be compared statistically^25^. Divergence of dog relative to wolf trajectories indicates heterotopic modification. Collinearity or parallelism of wolf and dog trajectories in shape space is a precondition to analyse heterochronic modifications. These manifest themselves as shifts of stage-specific locations (such as neonate, adult) along the shared trajectory direction.

In comparing skull shape modifications during domestication, it is crucial to take into account that past and recent processes of domestication and selection may be fundamentally different. In its early phase, the domestication process was likely driven by (relaxed) natural selection in an anthropogenic environment and unintentional artificial selection, probably for tameness^26–28^. In contrast, cranial proportions of modern domestic dog breeds are clearly the effect of intentional artificial selection driven by aesthetic or functional requirements (e.g., refs^29,30^). One example is brachycephaly and airorhynchy, which describe a short skull relative to its width and a dorsally rotated rostrum in e.g., bulldogs^31^. Although initially described as the retention of juvenile characters (paedomorphosis)^12^, such extreme morphology is clearly neomorphic^15^ and likely related to alterations in the branchial arch developmental system^32,33^. To avoid the confounding effects of modern breeding regimes, we investigate dog breeds that are either relatively close to the wolf skull morphology (mesaticephalic), or were likely less subject to a strong artificial selection regime, such as pre-modern and archaeological domestic morphotypes.

‘Modern’ breeds are recognized by kennel clubs and have been subject to exhaustive breeding regimes with strict aesthetic requirements and closed bloodlines during the last 150 years or so^34^. This group is represented here by one breed that is wolf-like in appearance and size, the German shepherd (Table 1). ‘Premodern’ domestic dogs are defined here as populations that are geographically and/or culturally isolated from the modern domestic dog breeds and occupy well-supported basal positions on molecular phylogenetic trees^34–36^. This group is represented by the Afghan hound, Akita, Australian dingo, and New Guinea singing dog (Table 1). The latter two are feral^37^ and likely submitted to less intense artificial selection and more intense natural selection^38^. The premodern sample also includes a group from the early 19^th^ century, when dogs were bred and classified according to their function and selection for aesthetics traits was of minor importance. This study contains one such type of dog, the pointing dog (‘Hühnerhund’), which was used to track down prey during a hunt (Table 1). ‘Archaeological’ domestic dogs represent prehistoric (Neolithic) and proto-historic (Iron Age) dogs from Switzerland (Table 1)^39^; these reflect the most ancestral forms in our dataset.

**Table 1:** Number (N) of examined domestic dogs, grouped according to time periods (see text), and wolves.

Groups	N (adult)	N (juvenile)
**Ancestor**		
Wolf*	24	15
**Archaeological dogs**		
Iron Age and Neolithic turbary dogs	6	—
**Premodern dogs**		
Australian dingo*	7	6
Afghan hound*	6	2
Akita*	11	4
New Guinea singing dog	8	1
Pointing dog*	4	4
**Modern breed**		
German shepherd*	10	2

Groups and breeds which have been included into the trajectory analysis are indicated with asterisks.

We use this sample to address two questions: 1) when during ontogeny do the unique (neomorphic) features of the cranium of dogs appear? 2) Taking into account neomorphic differences between wolf and dogs, is there evidence for a paedomorphic pattern of cranial development in domestic dogs compared to wolves?

## Results

**Figure 1:**
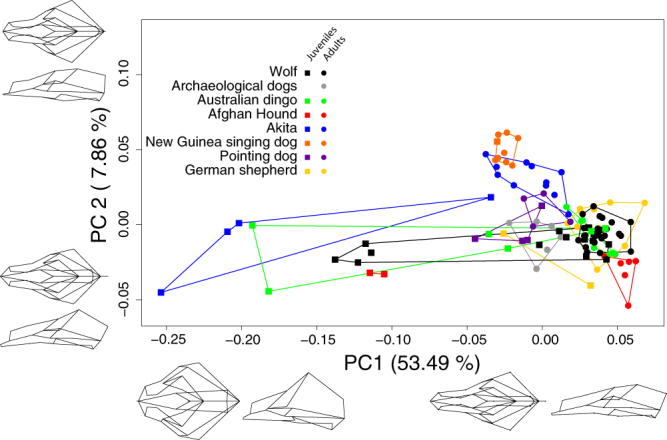
Cranial shape variation in ontogenetic series of domestic dogs and wolves. Groups are subdivided into juveniles and adults (all permanent teeth fully erupted into occlusion), as indicated by different shapes of data points and convex hulls. Wireframes of crania (for details see Fig. 4) represent extreme shapes on principal components (PC) 1 and 2. Ontogenetic changes are mainly along PC1, whereas PC2 indicates group/breed specific changes.

Figure 1 visualizes patterns of cranial shape variation along the first two principal components (PCs) of shape space. PC1 explains 53.49% of the total variation and is associated with a relative elongation and narrowing of the rostrum and a dorsoventral flattening of the braincase (Fig. 1). PC2 (7.86%) captures variation in rostrum width, neurocranial height, and rotation of the rostrum relative to the cranial base (Fig. 1). PC1 and 2 are further both associated with variation in the caudal protrusion of the external occipital protuberance (Fig. 1). The ontogenetic trajectories of cranial shape change in wolf and domestic dog extend mainly along PC1, whereas differences among groups/breeds of domestic dogs are mainly along PC2 (Fig. 1 **)**. However, there is overlap between groups along PC1 and PC2.

**Figure 2:**
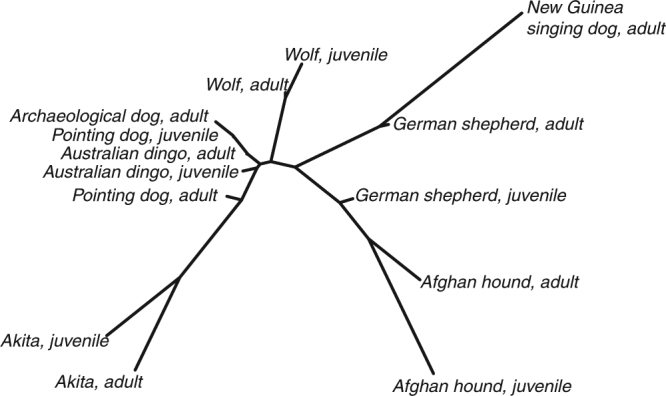
Neighbour joining tree of Mahalanobis distances showing skull shape differences among the investigated domestic dog groups/breeds and wolf and age stages.

Statistical comparisons of between-group morphological similarity (assessed by MANOVAs and quantified by Mahalanobis distances, Supplementary Information Table S1) show that the cranial morphology of domestic dogs not only differs from that of wolves during adulthood, but already in the juvenile forms (Fig. 2, Supplementary Informa﻿tion Table S1). The New Guinea singing dog is morphologically more distant from the wolf than most other domestic groups, including the modern German shepherd (Figs 1, 2, Supplementary Information Table S1). The archaeological dogs, pointing dogs and Australian dingos cluster together and are relatively closer to the wolves than the other domestic dogs (Fig. 2, Supplementary Information Table S1).

**Figure 3:**
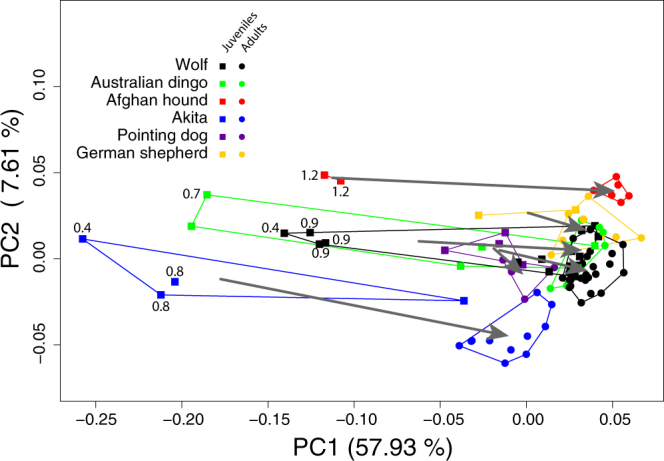
Ontogenetic trajectories of cranial shape in domestic dogs and wolves. Groups are subdivided into juveniles and adults (all permanent teeth fully erupted into occlusion), as indicated by different shapes of data points and convex hulls. The numbers associated with some data points indicate the absolute age of the corresponding specimens in months. The ontogenetic trajectories of wolf and domestic dogs are similar in direction and the trajectory of the Akita and Afghan are longer than in wolves (see Table 2).

Phenotypic trajectory analysis reveals that no difference exists between the direction of the wolf ontogenetic trajectory and that of any of the domestic dog groups/breeds (Table 2). However, the trajectories of wolf and dog breeds show differences in their position in morphospace (indicative for heterotopy), as well as in their length (indicative for heterochrony, Fig. 3, Table 2). Therefore, trajectories are largely parallel to each other and group-specific, heterotopic differences arise early during development, and persist into adulthood (Fig. 3). Given that group-specific trajectories are largely parallel to each other, it is possible to compare them in terms of heterochronic modification of their shared ancestral (wolf) trajectory. Compared to adult wolves, the adults of some dog groups are less advanced along the shared trajectory vector, i.e., have clearly paedomorphic crania (e.g., Australian dingo; Figs 1, 3). Others have similarly advanced cranial shapes (German shepherd) or even tend to be more advanced than wolves (peramorphic; Afghan hound; Figs 1, 3). For comparisons of trajectory length, the Akita sample is crucial because the youngest known-age specimens are of similar age as the youngest known-age wolf specimens (12 and 13 days, respectively, Fig. 3). The juvenile-to-adult trajectory of the Akita is slightly longer than that of the wolf (Fig. 3, Table 2, Supplementary Information Fig. S1). Most significantly, Akita juveniles have clearly more paedomorphic cranial shapes than wolf juveniles of the same age; Supplementary Information Fig. S1).

**Table 2:** Direction (pairwise vector correlations among first principal components) and distances (pairwise comparisons of vector length) of ontogenetic trajectories of skull shape change in domestic dog groups/breeds compared to the wolf.

Group/breed	Pairwise correlations	p-value	Path distances	p-value
Afghan hound	0.86	0.37	0.17	0.001
Akita	0.84	0.09	0.18	0.001
Australian dingo	0.89	0.18	0.1	0.13
German shepherd	0.57	0.05	0.07	0.81
Pointing dog	0.52	0.05	0.05	0.67

## Discussion

There is an overall similar direction of postnatal cranial ontogenetic trajectories in wolves and domestic dogs (Fig. 3, Table 2), implying that spatial patterns of cranial shape change are similar in these groups. We infer that the postnatal cranial ontogenies of wolf and domestic dogs follow a shared ancestral pattern of morphological change, but with heterochronic changes. On the other hand, skull shapes of domestic dogs and wolves are different already early after birth (Figs 1, 2, 3, Supplementary Information Table S1). Thus, we deduce that the group-specific neomorphic deviation of domestic dog crania from wolf crania likely reflects neomorphic processes during pre- and/or perinatal development. The ontogenetic timing of artificial selection might have an impact on the observed neomorphy early during postnatal ontogeny, as selection on domesticated animals is essentially a two-stage process: ‘culling’ of puppies for non-reproduction, followed by selection of adults for reproduction. Both processes might have had natural and cultural (intentional breeding) components.

In sum, our results provide support for both the neomorphosis hypothesis^12–15^ and the paedomorphosis hypothesis^5–10^, but at different stages of ontogeny. These patterns of ontogenetic modification are apparently not the result of recently applied artificial selection related to modern breed formation but were probably already present in early phases of the domestication process as a result of (relaxed) natural selection in an anthropogenic environment and/or ancient artificial selection (for tameness and/or increased reproduction rates)^26,28^.

While it is generally assumed that the cranial morphology of adult dogs is a good indicator of ongoing domestication and related (artificial) selection regimes^40–43^, our ontogenetic data allow us to critically assess this supposition. Adult archaeological dogs, pointing dogs, and Australian dingos, with a supposedly more ancestral skull conformation, are indeed more similar to adult wolves than groups which have been artificially selected more intensively, such as Akita, Afghan hound, and German shepherd (Figs 1, 2, Supplementary Information Table S1). On the other hand, adult New Guinea singing dogs, which are unlikely to have been subject to strong artificial selection, occupy positions in cranial shape space that are more distant from adults of wolves than any other domestic dog group/breed (Figs 1, 2). We conclude that adult skull shape alone cannot be used as a reliable indicator of paedomorphic processes during dog domestication, nor of specific domestication-related selection regimes. (The term ‘paedomorphosis’ is typically only used to compare adults – i.e., endpoints of ontogenetic trajectories – of ancestral and descendant groups. Here we use the term to describe ancestor-descendant differences between same-age groups at any point along the ontogenetic trajectory). However, our data on juveniles indicate a previously undescribed mechanism of paedomorphic shift during early ontogeny that might be relevant to understand dog domestication. As shown in Figure 3 and Supplementary Information Figure S1, known-age domestic dog juveniles are consistently more paedomorphic than wolves at the same age. Gestation length is relatively constant in different-sized domestic dogs^44,45^ and remarkably invariant between the domestic dog (64–66 days^46^) and the wolf (60–65 days^47^). In view of these data, the developmental shifts documented here (Fig. 3) likely indicate a pattern of deceleration of the rate of pre/perinatal skull development in dogs compared to wolves, resulting in a paedomorphic shift during early postnatal development. Depending on the rate of postnatal ontogeny, paedomorphic neonates might result in paedomorphic adults, in wolf-like adults (neutral in terms of heterochrony), or even in peramorphic adults. The known-age juvenile subsample available for this study is comparatively small, and larger samples will be required to substantiate the hypothesis of early paedomorphic shifts.

A trend toward more altricial newborns would present the ideal neurological substrate for the development of behavioral features such as tameness. It has been hypothesized that a low degree of organ differentiation at birth may lead to a more flexible reaction of the development to environmental conditions after birth (e.g., through artificial selection) because organogenesis (e.g., bone formation, brain development) is still underway^48^ and the development of the brain might be more sensitive to environmental influences (such as social context). Indeed, it has been shown that in silver foxes that had been selected for tameness over 45 generations, and in domestic dogs the period of socialization begins earlier than in their non-tamed/wild ancestors (see ref.^49^ for a review).

The ‘early paedomorphosis’ hypothesis offers interesting comparative perspectives on the proximate, developmental patterns of domestication. Humans compared to our closest relatives, the great apes, are characterized by secondary altriciality (helpless, fetal-like neonates). A trend toward secondary altriciality has long been postulated to be a prerequisite of early cognitive and social development in humans and their fossil relatives^50^, as early brain development is strongly influenced by an individual’s social environment.

To conclude, we found that the process of dog domestication involved evolutionary developmental modifications that resulted in a combination of neomorphic and paedomorphic features of cranial morphology. Hypotheses about paedomorphic shifts cannot be answered by comparing adult morphotypes alone, but require complete comparative ontogenetic series. Ideally, investigations on cranial shape changes during perinatal development of domestic dogs and wolves will further elucidate the significance of early developmental shifts during domestication.

## Materials and Methods

### Specimens, landmarks, and age stages

Ontogenetic series of crania of 110 specimens were used in this study: 39 wolves (15 juveniles, 24 adults) and 71 domestic dogs (19 juveniles, 52 adults) (Table 1). The specimens are housed in the Zoologisches Institut/Populationsgenetik (former Institut für Haustierkunde), Christian-Albrechts-Universität zu Kiel, Germany (I.f.H.); Naturhistorisches Museum Bern, Switzerland (NMBE); Naturhistoriska Riksmuseet, Stockholm, Sweden (NRM). One CT-scanned specimens is housed in the Zoologisches Institut und Museum der Universität Zürich, Switzerland (ZMUZH) and three CT-scanned specimens were contributed from the Tierpark Bern, Dählhölzli & Bärenpark, Switzerland (TBDB). No specimen was killed for this study and no live specimens were used.

**Figure 4:**
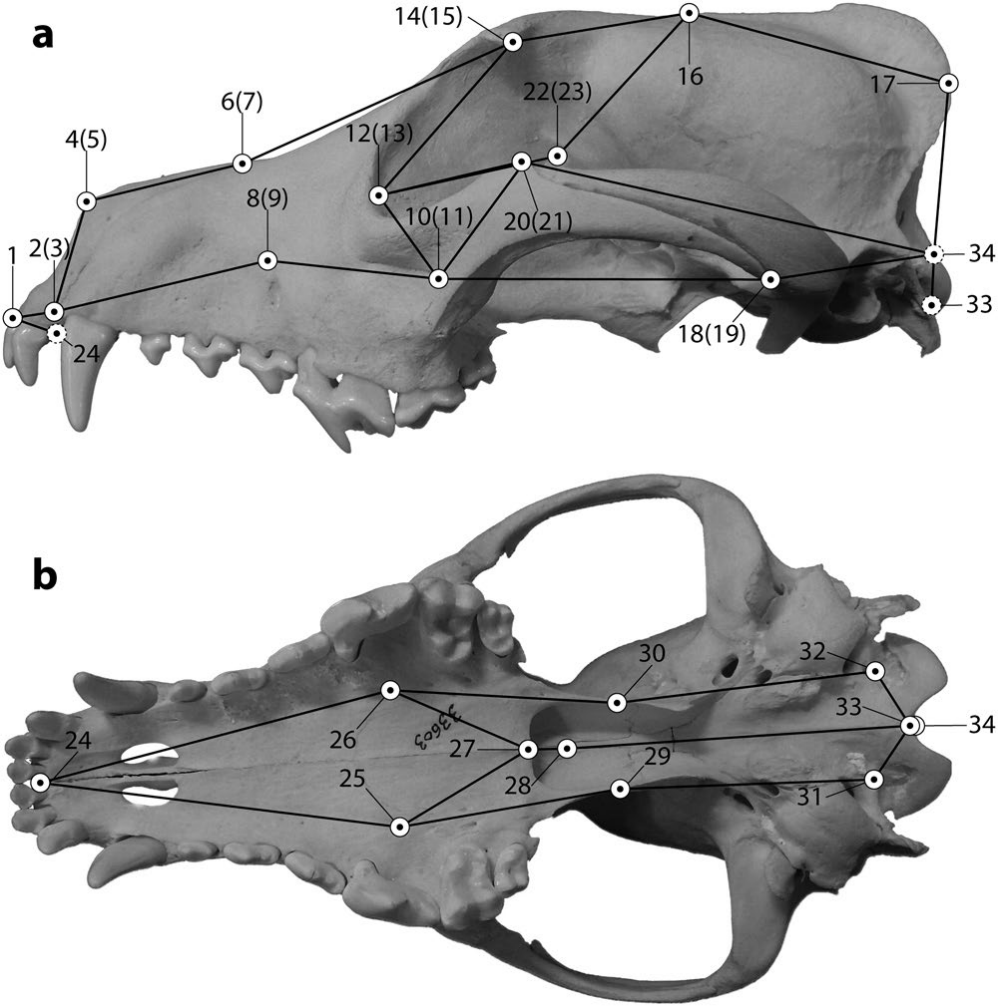
Position of landmarks used in this study. The landmarks are displayed on a skull of an Australian dingo in lateral (**A**) and ventral (**B**) aspects. Numbers of landmarks correspond to Table 3 and numbers in brackets refer to the respective contralateral landmark. Connections between landmarks represent the wireframe that was used to illustrate shape changes along principal components (Fig. 1). Dashed lines indicate landmarks that are not visible in the respective aspect.

The three-dimensional coordinates of 34 landmarks on the left and the right side of the skull (Fig. 4, Table 3) were captured with a MicroScribe MX 3D digitizer (Solution Technologies, Inc.). Additionally, one specimen was scanned with a micro-CT (XtremeCT II, SCANCO Medical AG, Brüttisellen, Switzerland) and three specimens were scanned with a medical CT (SOMATOM Definition Flash, Siemens Healthcare, Forchheim, Germany). Isosurfaces were subsequently extracted with Avizo version 6.2.1 or VGStudio MAX version 2.2.5 and landmarks were captured with MeshLab version 1.3.3. The choice of landmarks was based on their recognizability in all age stages. The ventral and the dorsal aspects of the crania th﻿at have been digitized ﻿with a MicroScribe were combined into one set of coordinates for the entire skull through digitizing three landmarks from both aspects. Forty-nine crania were digitized twice to account for random errors in individual measurements during digitization. To determine the amount of measurement error between the two replicates in every specimen, Procrustes ANOVA was calculated^51^. The amount of shape variation due to digitizing error in the skull is 58–117 times smaller than any biological variation (among individuals and/or between the two sides of the skull) and is thus considered negligible. Captive wolves were only considered if they were less than one month old because skull shape can be greatly altered in captive specimens^52,53^. However, these changes were not considered extensive in very young specimens. All scanning data of the specimens using the medical CT are available in MorphoMuseuM^54^.

**Table 3:** Numbers and definition of the used landmarks. No., landmark number as depicted in Fig. 4.

No.	Cranial landmarks
1	Dorsal interpremaxillary suture, inferiormost point of the bony septum between the upper central incisors (dorsal)
2	Premaxillary-maxillary suture, inferiormost point, left side (dorsal)
3	Premaxillary-maxillary suture, inferiormost point, right side (dorsal)
4	Nasal bone, anterior tip, left side (dorsal)
5	Nasal bone, anterior tip, right side (dorsal)
6	Intersection of nasal, maxillary, and frontal bones, left side (dorsal)
7	Intersection of nasal, maxillary, and frontal bones, right side (dorsal)
8	Infraoribtal foramen, superiormost point, left side (dorsal)
9	Infraoribtal foramen, superiormost point, right side (dorsal)
10	Zygomatic process of maxilla, posterior tip, left side (dorsal)
11	Zygomatic process of maxilla, posterior tip, right side (dorsal)
12	Fossa for lacrimal sac, inferior margin, left side (dorsal)
13	Fossa for lacrimal sac, inferior margin, right side (dorsal)
14	Zygomatic process of frontal bone, tip, left side (dorsal)
15	Zygomatic process of frontal bone, tip, right side (dorsal)
16	Bregma, intersection of interfrontal, interparietal, and frontoparietal sutures (dorsal)
17	Inion, highest projection of the external occipital protuberance (dorsal)
18	Jugo-squamosal suture, inferior intersection, left side (dorsal)
19	Jugo-squamosal suture, inferior intersection, right side (dorsal)
20	Zygomatic process, highest projection, left side (dorsal)
21	Zygomatic process, highest projection, right side (dorsal)
22	Dorsal ethmoidal foramen, inferior margin left side (dorsal)
23	Dorsal ethmoidal foramen, inferior margin, right side (dorsal)
24	Ventral interpremaxillary suture, anteriormost point of the bony septum between the upper central incisors (ventral)
25	Major palatine foramen, posterior margin, right side (ventral)
26	Major palatine foramen, posterior margin, left side (ventral)
27	Interpalatine suture, posteriormost point (ventral)
28	Presphenoid, anterior tip (ventral)
29	Palatine-pterygoid suture, inferiormost edge, right side (ventral)
30	Palatine-pterygoid suture, inferiormost edge, left side (ventral)
31	Hypoglossal canal, posterior margin, right side (ventral)
32	Hypoglossal canal, posterior margin, left side (ventral)
33	Basion, ventral margin of the foramen magnum (ventral)
34	Opisthion, dorsal margin of the foramen magnum (ventral)

A few specimens were not complete, i.e., not all landmark positions were measurable. There were 21 specimens with at least one missing landmark. The maximum number of missing landmarks in one specimen was 4 (a juvenile Australian dingo). The missing landmarks were estimated using the ‘estimate.missing’ function in the geomorph-package version 2.1.4 in R. This function interpolates the coordinates of a landmark of a reference specimen (obtained from a set of specimens for which all landmarks are present) on the specimen with missing landmarks using the thin-plate spline.

All wolf and domestic dog specimens were allocated to a dental age stage: if all permanent teeth were fully erupted into occlusion, specimens were described as adults. If deciduous and not jet fully erupted permanent teeth were still present, the specimens were described as juveniles. For the wolf and every group/breed of domestic dog, ontogenetic series were sampled. This was, however, not possible for the archaeological specimens. The youngest domestic dog (Akita) and wolf are comparable in absolute age (12 and 13 days old). On the other extreme, domestic dog and wolf specimens of more than 10 years of age have been sampled.

### Analyses

All analyses were conducted using Microsoft Excel 2010, R version 3.2.1^55^ and RStudio version 1.0.136^56^. The mean of both replicates was calculated for every landmark in all specimens that have been digitized twice. The complete dataset is available as part of the supplementary data (Supplementary Table S2).

A generalized Procrustes superimposition was computed to extract geometric shape of the investigated specimens simultaneously. Subsequently, a covariance matrix of the symmetric component of averaged Procrustes shape coordinates (average of the left and right sides of the skull) was generated. Based on this covariance matrix, a principal component analysis (PCA) was performed to explore patterns of shape variation in domestic dogs and wolves. Differences between groups and age classes in skull shape were assessed with MANOVAs and quantified using Mahalanobis distances. P-values from MANOVAs were corrected for multiple testing.

Phenotypic ontogenetic trajectories of wolves and domestic dogs in shape space were compared^57,58^ using the ‘trajectory.analysis’ function in the geomorph-package version 2.1.4^59^ in R. In doing so, cranial shape changes from juveniles to adults were quantified and compared between wolf and groups/breeds of domestic dogs indicated in Table 1. This analysis compares the trajectory size defined as the path-length distance along the trajectory, and the trajectory orientation (direction) defined as the direction of first principal component of the covariance matrix estimated from the trajectory points after standardisation for the starting point^21^. A permutation procedure (1000 random permutations) was used to statistically test differences of sampling distributions.

### Data availability

All data generated or analysed during this study are included in this published article (and its supplementary information files).

## Electronic supplementary material

Supplementary Information

Supplementary Table S2
