# Supplementary material for: Neomorphosis and heterochrony of skull shape in dog domestication
Source: Sci Rep. 2017 Oct 18;7:13443. doi: 10.1038/s41598-017-12582-2 (PMC5647425; doi:10.1038/s41598-017-12582-2)
Supplement: Supplementary file 1 — Supplementary Information [file 41598_2017_12582_MOESM1_ESM.pdf]

Neomorphosis and heterochrony of skull shape in dog domestication

Madeleine Geiger, Allowen Evin, Marcelo R. Sánchez-Villagra, Dominic Gascho, Cornelia Mainini, Christoph P. E. Zollikofer

Supplementary Figure S1. Visualization of the relationship between the log of absolute age of the specimens (in days) and the log (centroid size) (top), and PC1 (bottom). The grey shaded area signifies the age range of dental maturity, which commences at on average five months in domestic dogs and wolves[1]. Note that very young juveniles of Akita and Australian dingo have smaller cranial size (top) and less advanced cranial shape (bottom) than wolves of similar age.

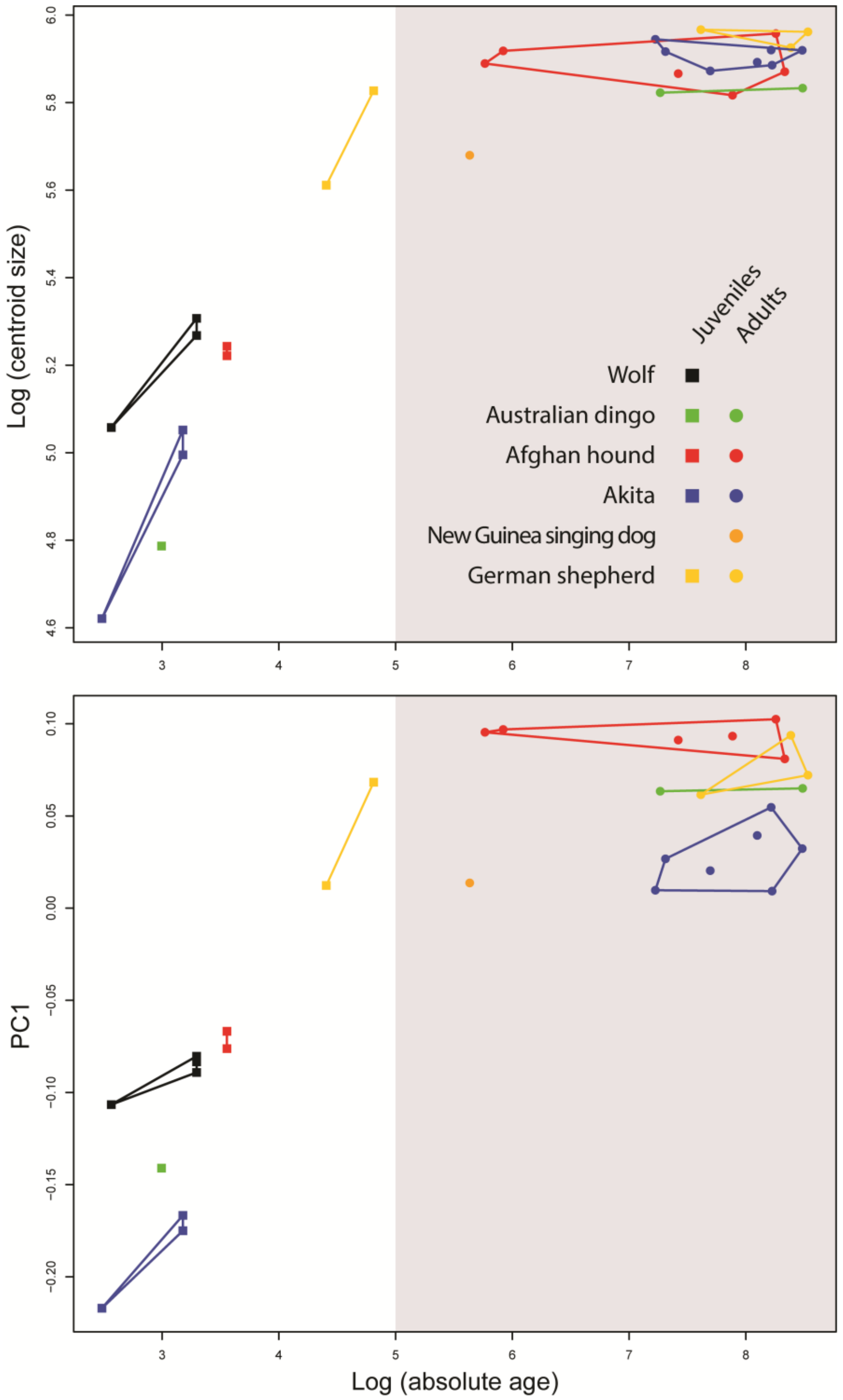

Supplementary Table S1. Mahalanobis distances and associated p-values from all domestic dog groups and the wolf. Distances are given below and p-values from pairwise MANOVA above the diagonal. Significant p-values are indicated in bold if they remained significant subsequent to corrections for multiple testing. Ad, adult; Afgh, Afghan hound; Akit, Akita; Ausd, Australian dingo; Gers, German shepherd; juv, juvenile; Ngsd, New Guinea singing dog; Prhd, Prehistoric dogs; Poid, Pointing dog.

	Afgh juv	Afgh ad	Akit juv	Akit,ad	Ausd juv	Aus ad	Gers juv	Gers ad	Ngsd ad	Prhd ad	Poid juv	Poid ad	Wolf juv	Wolf ad
Afgh juv		**<0.0001**	0.37	**<0.0001**	0.33	**<0.0001**	0.07	**<0.0001**	**<0.0001**	**<0.0001**	**<0.0001**	**<0.0001**	**<0.0001**	**<0.0001**
Afgh ad	41.91		**<0.0001**	**<0.0001**	**<0.0001**	**<0.0001**	**<0.0001**	**<0.0001**	**<0.0001**	**<0.0001**	**<0.0001**	**<0.0001**	**<0.0001**	**<0.0001**
Akit juv	59.49	111.17		**<0.0001**	**0.04**	**<0.0001**	0.23	**<0.0001**	**<0.0001**	**0.01**	0.07	0.05	**<0.0001**	**<0.0001**
Akit,ad	102.36	82.79	48.14		**<0.0001**	**<0.0001**	**<0.0001**	**<0.0001**	**<0.0001**	**<0.0001**	**<0.0001**	**<0.0001**	**<0.0001**	**<0.0001**
Ausd juv	36.16	29.73	46.92	37.06		**0.02**	0.63	**<0.0001**	**<0.0001**	**<0.0001**	**<0.0001**	**0.03**	**0.01**	**<0.0001**
Aus ad	67.38	25.10	72.10	35.73	13.51		**<0.0001**	**<0.0001**	**<0.0001**	**<0.0001**	**<0.0001**	**0.01**	**<0.0001**	**<0.0001**
Gers juv	26.05	27.26	72.89	76.79	18.18	40.10		**0.01**	**<0.0001**	**<0.0001**	0.12	0.09	**0.03**	**<0.0001**
Gers ad	46.05	18.06	104.02	67.48	32.81	43.55	30.83		**<0.0001**	**<0.0001**	**<0.0001**	**<0.0001**	**<0.0001**	**<0.0001**
Ngsd ad	91.12	76.82	83.42	43.38	59.76	47.97	100.98	66.52		**<0.0001**	**<0.0001**	**<0.0001**	**<0.0001**	**<0.0001**
Prhd ad	77.09	32.60	83.25	46.72	12.40	10.78	31.60	42.39	73.32		**0.01**	**0.02**	**<0.0001**	**<0.0001**
Poid juv	49.49	37.54	60.13	46.10	8.70	24.03	10.61	35.64	76.69	10.67		0.07	**<0.0001**	**<0.0001**
Poid ad	64.07	33.85	65.33	23.22	12.43	17.15	28.77	25.49	48.32	10.51	10.12		**<0.0001**	**<0.0001**
Wolf juv	54.35	39.44	75.52	60.36	15.55	25.49	35.85	35.25	78.08	25.67	27.61	27.17		**<0.0001**
Wolf ad	70.20	33.61	78.79	41.62	19.50	16.14	45.86	29.85	63.43	22.56	32.47	20.43	6.44	

Supplementary references

1. Geiger, M., Gendron, K., Willmitzer, F. & Sánchez-Villagra, M. R. Unaltered sequence of dental, skeletal, and sexual maturity in domestic dogs compared to the wolf. *Zool. Lett.* **2,** 16 (2016).
